# The Effects of a Strategic Instructional Self-Talk Intervention on Performance in a Complex Tennis Rally

**DOI:** 10.3390/bs16010087

**Published:** 2026-01-08

**Authors:** Evangelos Galanis, Polydoros Kouvarakis, Olga Kouli, Charalampos Krommidas, Nikos Comoutos, Antonis Hatzigeorgiadis, Yannis Theodorakis

**Affiliations:** 1 Department of Physical Education and Sport Science, University of Thessaly, 421 00 Trikala, Greece; egalanis@uth.gr (E.G.); polydoroskouvarakis@gmail.com (P.K.); hkrom@uth.gr (C.K.); nzourba@uth.gr (N.C.); ahatzi@uth.gr (A.H.); 2 Department of Physical Education and Sport Science, Democritus University of Thrace, 691 00 Panepistimioupoli, Greece; okouli@phyed.duth.gr

**Keywords:** self-talk mechanisms, shifting attention, tennis strokes, self-regulation, performance enhancement

## Abstract

The purpose of the present study was to examine the effects of a strategic self-talk intervention on a complex tennis performance test through the use of a narrow internal instructional self-talk plan. Fifty young beginner tennis players from two tennis academies were assigned into intervention and control groups. A pre/post quasi-experimental design was implemented, including baseline assessment, training intervention, and final assessment. The intervention lasted five weeks, during which all participants underwent the same training, with the experimental group using strategic self-talk. Repeated measures MANOVA revealed a significant time by group interaction for all tennis strokes; pairwise comparisons showed that the all strokes performance of the self-talk group improved from initial to final assessment and was better than the performance of the control group at final assessment. In addition, repeated measures ANOVA examining the overall performance of the rally showed that the intervention group achieved higher total rally scores than the control group. The effectiveness of the intervention in this multi-stroke task suggests that strategic self-talk facilitated movement sequences, possibly through an efficient shifting of attention. Accordingly, practitioners are encouraged to explore the potential of strategic self-talk for tasks requiring such attentional demands.

## 1. Introduction

The applied value of self-talk strategies in sports contexts has been thoroughly documented in the self-talk literature (Hardy et al., 2018; Hatzigeorgiadis et al., 2020; Latinjak et al., 2023). According to a recent conceptualization of self-talk (Latinjak et al., 2019), the strategic form of self-talk aims to enhance sports performance by using predetermined and practiced cue words to activate appropriate responses (Hatzigeorgiadis et al., 2014a). Previous research has highlighted the effectiveness of strategic self-talk interventions through various experimental designs (e.g., controlled trials, crossover designs), populations (e.g., pupils/students, young and beginner athletes, and elite athletes), tasks (e.g., fine and gross motor/sport skills), sport types (e.g., individual and team), and settings (e.g., laboratory and field). More specifically, two reviews, a meta-analysis (Hatzigeorgiadis et al., 2011) and a systematic review (Tod et al., 2011), have provided robust support for the beneficial effects of strategic self-talk on skill acquisition, learning, and sports task performance.

An important determinant for the effectiveness of self-talk is the use of appropriate types of cues, which trigger suitable responses, depending on parameters such as the task type and level of expertise; this assumption that has been termed the matching hypothesis (Hardy et al., 2009). Instructional self-talk cues, aiming to provide direction for action, are more suitable for attentional purposes (e.g., focusing attention, improving concentration, and directing attention) compared with motivational cues; therefore, they are more beneficial for relatively fine tasks and for the earlier stage of learning. In turn, motivational self-talk cues, aiming to increase drive, are more suitable for motivational purposes (e.g., enhancing confidence, regulating effort) compared with instructional cues; therefore, they are more beneficial for relatively grosstasks and for more advanced levels of expertise (Hatzigeorgiadis et al., 2014b).

Taking into consideration the facilitating effects of strategic self-talk on sports task performance, as well as the matching hypothesis, contemporary research has shifted toward exploring the mechanisms that explain the facilitating effects of strategic self-talk on sports task performance (Galanis & Hatzigeorgiadis, 2020). Research on self-talk mechanisms has been mostly focused on two broad clusters of mechanisms mediating the effect of strategic self-talk on performance: attentional and motivational (Galanis et al., 2016). Hatzigeorgiadis and Galanis (2017), reviewing evidence regarding attentional mechanisms, postulated that strategic self-talk can enhance attentional focus, shift attention in the appropriate attentional style or direct attention to the appropriate stimuli, and improve attentional functions. Subsequently, they suggested that the effects of strategic self-talk on attention are, in part, responsible for the effectiveness of strategic self-talk. To further explore such assumptions, recent studies have examined the effects of strategic self-talk on attention-demanding tasks under attention-threatening conditions. The results have shown that strategic self-talk facilitated performance under conditions of external distraction (Galanis et al., 2018), ego depletion (Gregersen et al., 2017), and physical exertion (Galanis et al., 2022a), thus reinforcing the attentional impact of strategic self-talk for sport performance.

An aspect of attention that has attracted considerable research in sport is the differentiation between internal and external focus of attention. According to Nideffer’s (1976) attentional style model, an internal focus directs attention towards one’s own body movements (e.g., a stretched arm), while the external focus directs attention towards movements in the environment (e.g., a tennis ball). Alongside internal–external attention focus, the width of focus specifies whether it involves narrow (e.g., very few specific stimuli) or broad (e.g., many stimuli) attention. In a study on golf performance, Bell and Hardy (2009) examined the impact of attention on skilled performance using three focus cues: internal, external, and distal external. Under two conditions—neutral and anxious—the results showed a better performance with the distal external focus than internal or proximal external in both conditions.

Tennis is a sport with high attentional demands for the acquisition and development of the technical components of execution, for attaining and maintaining focus on continuous ongoing stimuli, and regaining attentional aspects after small intervals (Cece et al., 2020), often under attention-threatening conditions. Several studies have explored the effects of strategic self-talk on aspects of tennis performance, such as serve, forehand drives, and volleys. Malouff et al. (2008) examined the effects of self-instruction on serving accuracy in a tennis serving competition. The results showed that the self-instruction group served significantly more accurately than those in the control group. Cutton and Landin (2007) compared the effects of strategic instructional self-talk and teacher-provided knowledge of performance feedback on learning the forehand tennis groundstrokes in a class setting. The results showed that the strategic instructional self-talk group performed better in the movement sequence than the feedback group. Similarly, Latinjak et al. (2011) found that tennis players who used regular strategic instructional self-talk or a combination of strategic instructional self-talk and self-feedback concentrated more effectively and performed better in forehand groundstrokes than the control group. Finally, Landin and Hebert (1999) reported improvements in movement patterns and stroke accuracy among collegiate tennis players in a study examining the effects of two sequential strategic instructional cues on volleying skill.

Mostly related to our research interests, in one of the first studies exploring the potential of strategic self-talk, Ziegler (1987) examined the effects of self-talk cues on tennis groundstrokes among beginner tennis players, adopting a narrow external mode of attentional self-talk instruction. Specifically, the participants were instructed to focus their attention on the appropriate stimuli following four self-talk cues: (a) “ball”—focusing on the early tracking of the ball, (b) “bounce”—focusing on the pathway of the ball, (c) “hit”—focusing on ball contact, (d) “ready”—focusing on the preparation for the next ball. The results revealed a significant improvement in forehand and backhand performance, and the author postulated that the players improved their ability to focus attention on the ball.

Considering an interacting approach regarding the value of internal and external focus, it has been argued that an external focus would be more beneficial for skilled/expert athletes who have reached the level of atomization, whereas an internal focus would be beneficial for less skilled athletes in the learning stages (Bernstein, 1996). Empirical support for the beneficial effects of internal focus of attention among beginners has been provided by Beilock et al. (2002) who subsequently argued that different cognitive processes are required for people at different levels of skill acquisition.

In light of the existing literature, we aimed to progress research by targeting two sport-related aspects, the difficulty of the sport task and attentional demands. Regarding the difficulty of the task, research in the strategic self-talk literature has almost exclusively focused on simple motor (e.g., Edwards et al., 2008; Kolovelonis et al., 2011) and sport (e.g., Abdoli et al., 2018; Yang et al., 2024) tasks. Accordingly, studies on tennis (as described above) have also targeted isolated tennis strokes, such as the serve, groundstrokes, and volleys. Regarding the exploration of the attentional mechanism, research has examined different aspects of attention such as attentional focus (e.g., Sarig et al., 2023), different dimensions of attention (selective, divided, spatial) through computerized tasks (Galanis et al., 2022b), and tasks under attention-threatening conditions (Galanis & Hatzigeorgiadis, 2020). Taking into account these parameters, to advance research in the relevant area and approach a more realistic match situation, while at the same time augmenting the attentional demands of the tennis task and involving the aspect of shifting attention, a multi-stroke tennis rally including serve, groundstrokes, and volleys was employed as the performance outcome.

Based on the above evidence regarding the different attentional functions of self-talk, our study aimed to explore the effects of a narrow internal instructional self-talk intervention on tennis performance among young tennis players, taking attentional aspects into perspective. Identifying that in the tennis self-talk literature, performance has been assessed through isolated tennis strokes, and considering the attentional requirements of tennis, we focused on the attention-shifting function of self-talk. To achieve this goal, we envisaged a complex, multi-stroke tennis rally, including serve, groundstrokes, and volleys, which would increase the difficulty of the task, place emphasis on shifting attention, and enhance the ecological validity of the experiment. It was hypothesized that tennis players using strategic instructional self-talk will have better performances compared with the players of the control group.

## 2. Methods

### 2.1. Participants

Fifty young beginner tennis players (23 boys and 27 girls) from two tennis academies (twenty-five from each academy) with a mean age 13.01 (SD = 1.49) years were recruited for this study. The players had between one and two years of training experience and no previous competitive experience. They were familiar with the strokes involved in the assessment and they all had similar tennis skills. None of the tennis players had prior experience of any psychological skills training.

### 2.2. Apparatus

A Lobster Grand V ball machine was used to throw the balls (Wilson US open) at the tennis players from the baseline of the opposite side of the court. For each rally, the machine was adjusted to throw four balls (two for the groundstrokes and two for the volleys) at a speed of 55 mph, launched every two seconds; a medium trajectory (second among three levels of difficulty) was selected. The balls were set to land at the ¾ of the opposite court, right and left of the court’s center.

### 2.3. Tennis Performance Test

For the purposes of this study, Hensley’s Tennis Skills Test Manual (Hensley, 1989) was modified. Each tennis player was evaluated through a complex tennis rally, which simulated playing conditions including serve, two groundstrokes, and two volleys. In particular, participants started the tennis rally with a serve that was followed by a forehand and a backhand groundstroke; then they proceeded to the volley position, performing a forehand and a backhand volley. The test was repeated ten times, five serving from the right side of the court and five from the left. Thus, each tennis player received scores for a total of 10 serves, 10 forehand groundstrokes, 10 backhand groundstrokes, 10 forehand volleys, and 10 backhand volleys.

According to Hensley’s description, each tennis stroke has its own scoring points. The points for each stroke depended on the landing spot of the ball, with balls landing closer to the baseline or the sidelines giving more points. For the serve, the scoring ranges from 1 to 2 points; for the groundstrokes, from 1 to 4 points; and for the volley, from 1 to 4 points (the precise scoring can be found in the test manual, Hensley, 1989). To accurately record participants’ scores, three experienced judges were recruited, each one responsible for the three score areas. Two were placed on the court sides (one to judge groundstrokes and the other to judge volleys) and one was placed behind the baseline (to judge serves), next to the ball landing areas for a clearer view. Colored strips (blue, green, and red) were placed on the tennis court, with each color representing a different tennis stroke.

### 2.4. Procedure and Intervention

The institution’s ethics committee provided permission for the conduct of this study (ref: 1252). Coaches from two collaborating tennis academies were invited and agreed to take part in the study. The two tennis academies had a similar training schedule, with players training three days per week for one hour per day. The young tennis players were informed about the requirements of the study, which placed emphasis on the consistency of participation throughout the five weeks. Subsequently, they were asked to volunteer for the program. Twenty-five volunteers from each club (out of 35 and 70) were randomly selected to participate in the study. They were informed that the data would be confidential and that they could withdraw from the study at any time. Before the onset of the study, informed consent was signed by the participants’ parents. A quasi-experimental design was adopted. A cluster assignment of groups was used to avoid the contamination of the experimental conditions (allocation by academy). Subsequently, the two groups were randomly assigned as control and experimental. The coaches of the two clubs had long cooperated and agreed to provide the same training program during the five weeks. All participants attended the program consistently; none of the players missed more than two training sessions in the 5-week program. Participants of the two groups had a similar intervention training schedule and spent the same time practicing the experimental drills.

#### 2.4.1. Initial Assessment

All participants received information about the requirements and the procedures for the initial assessment. Subsequently, a 10 min regular warm up was applied. Finally, participants performed the 10 rallies. Each tennis player was tested individually and the whole procedure lasted approximately 60 min per day.

#### 2.4.2. Practice and Intervention

Following the completion of the initial assessment, players of both groups attended the experimental protocol, including one hour of practice twice per week during their typical training sessions for five weeks. In these training sessions, participants of both groups performed, at the beginning of the training, following their standard warm-up, one set of 10 repetitions for serves, forehand, and backhand groundstrokes, and forehand and backhand volleys, with a 2 min interval between the sets.

In the first training session, participants of the intervention group received a brief presentation on strategic self-talk and it was explained how the self-talk plans would be introduced into their practice. The self-talk plans were implemented under the supervision of the researcher for all training sessions. At the start of each session, participants received instructions about the self-talk plans; in particular, they were instructed on what to say (e.g., “legs”), when to say it (e.g., “just before the serve”), and why to say it (e.g., “to bend the legs”). The self-talk plans involved cue words that aimed to trigger narrow internal attention by using strategic instructional self-talk, preparing participants to focus on performing the strokes with the correct technique. More specifically, three cue words (“arm”, “legs”, and “core”) were used to fit with the tennis strokes (serves, groundstrokes, and volleys), with each one corresponding to a certain body posture the participant should have while performing each stroke. For example, for the serve, the cue word “arm” reflected the instruction to extend the arm to hit the ball at its highest point; for the groundstrokes, the cue word “legs” reflected the instruction to bend their knees to check the correct position for receiving the ball; and for the volley, the cue word “core” reflected the instruction to stretch the body to reach the ball at the optimal point. Players practiced the strategic self-talk cues separately in the initial sessions and in combination later in the intervention. Eventually, they were instructed to devise a self-talk plan to use for the final assessment rally. The use of the self-talk cues was verbalized out loud to ensure its use.

#### 2.4.3. Final Assessment

Following the completion of the intervention program, participants took part in the final assessment. The same procedures applied in the initial assessment were repeated, only this time, participants of the intervention group were asked to apply the strategic instructional self-talk cues that had been practiced during the intervention phase, in any combination that suited each player’s needs and preferences. The cues were verbalized out loud during the tennis performance test. Each participant was tested individually and the whole procedure lasted approximately 60 min per day.

At the end of the experiment, participants of the control group and players not drawn to participate in the study from both academies were offered a workshop about self-talk in tennis.

## 3. Results

### 3.1. Preliminary Analysis

Data screening showed that there were no missing data. The assumption for normality, homogeneity, and sphericity was met. One-way MANOVA was performed to examine performance differences in the three tennis strokes between the two groups at initial assessment. The results revealed a non-significant multivariate effect, *F*(3, 46) = 1.26, *p* = 0.30. Accordingly, the examination of the univariate effects showed no significant differences for the serve, *F*(1, 49) = 3.57, *p* = 0.07, for the groundstroke, *F*(1, 49) = 0.07, *p* = 0.79, and for the volley, *F*(1, 49) = 0.02, *p* = 0.90.

### 3.2. Main Analysis

A two-way (time × group) repeated measures ANOVA was performed to examine performance changes in the three strokes from the initial to the final assessment for the two groups. The results revealed a significant time by group interaction effect, *F*(3, 46) = 11.18, *p* < 0.01, *η*^2^ = 0.42. The examination of univariate effects showed a significant time by group interaction for all three strokes: for the serve, *F*(1, 48) = 9.93, *p* < 0.01, *η*^2^ = 0.17, for the groundstroke, *F*(1, 48) = 6.07, *p* < 0.05, *η*^2^ = 0.11, and for the volley, *F*(1, 48) = 12.98, *p* < 0.01, *η*^2^ = 0.21. Pairwise comparisons revealed that (a) the performance of the intervention group improved significantly for all three strokes, serve (*p* < 0.01), groundstroke (*p* < 0.01), and volley (*p* < 0.01), while there were no significant differences for the control group (*p* = 0.61, *p* = 0.17, and *p* = 0.08, respectively); (b) in the final assessment, the performance of the intervention group was superior to that of the control group for all strokes, serve (*p* < 0.001), groundstroke (*p* < 0.001), and volley (*p* < 0.001). The descriptive statistics for the three strokes are shown in Table 1. In addition, considering the marginally not significant baseline difference between the two groups for the serve, a one-way ANCOVA was performed, with the final score as the dependent variable and the baseline score as the covariate. The analysis showed a significant group effect, *F*(1, 49) = 6.27, *p* < 0.05, *η^2^* = 0.12.

**Figure 1:**
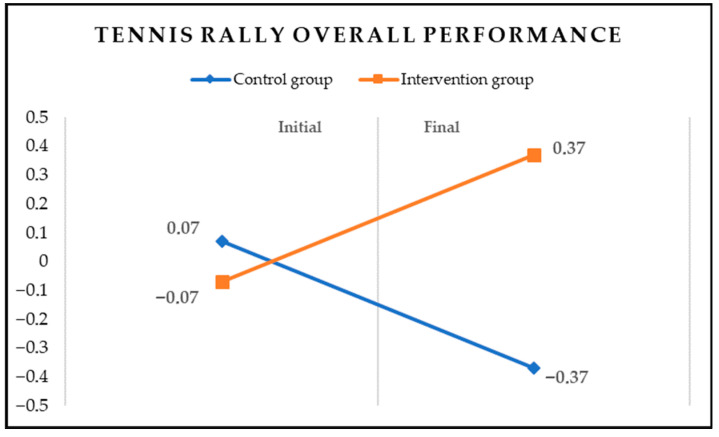
Tennis rally interaction effect for the two groups (Z scores).

To examine the differences in overall performance in the complex tennis rally, the score of each stroke was standardized (Z-score) and then the three scores were added together. A two-way (time x group) repeated measures ANOVA was performed to examine performance differences in the tennis rally between the initial and final assessments for the two groups. The results revealed a significant time by group interaction effect, *F*(1, 48) = 27.65, *p* < 0.01, *η*^2^ = 0.37, indicating that the intervention group had a significantly better performance than the control group (*p* < 0.01). The interaction effect for the overall rally performance is displayed in Figure 1.

## 4. Discussion

Strategic self-talk interventions have proven effective for enhancing sport task performance in a large variety of sports. Likewise, in tennis there are several studies that have supported the positive effect of strategic self-talk on isolated tennis strokes, such as the serve (Malouff et al., 2008) and the groundstrokes (Hatzigeorgiadis et al., 2009). Yet, attempts to examine the impact of strategic self-talk on tasks simulating playing conditions are lacking. In addition, considering the well-documented role of attentional processes justifying the facilitative self-talk effects on performance, a study combining different tennis strokes would, indirectly, provide indications regarding athletes’ attention-shifting skills. Accordingly, the present study examined the effectiveness of strategic self-talk, in the form of narrow internal instructional self-talk cues, on a complex tennis rally, involving basic tennis strokes: serve, groundstrokes, and volleys. Overall, the results showed that the use of strategic instructional self-talk improved performance for the different strokes and the overall rally performance, revealing medium to large effect sizes for the three strokes. The findings provide indications that instructional self-talk reinforcing an internal narrow focus facilitated the shifting of attention to sequential stimuli over the completion of the tennis rally.

The results of the present study are in line with the bulk of evidence supporting the effectiveness of self-talk in sport in general, and in tennis in particular. Prior studies examining isolated tennis strokes have documented that strategic instructional self-talk has produced improved performances in a serve competition among adult tennis players (Malouff et al., 2008) and in volley strokes among skilled female tennis players (Landin & Hebert, 1999). Furthermore, studies have examined, in addition to performance, the impact of strategic self-talk on secondary outcomes, as potential mechanisms explaining the effectiveness of self-talk. In particular, Hatzigeorgiadis and colleagues in experiments with young tennis players reported that enhanced performance following a strategic self-talk intervention was related with increased self-efficacy (Hatzigeorgiadis et al., 2008) and reduced cognitive anxiety and enhanced self-confidence (Hatzigeorgiadis et al., 2009). Such findings have contributed to the understanding of the mechanisms underlying strategic self-talk benefits.

Two studies have examined the effectiveness of multiple sequential self-talk cues on single tennis stroke performance. Ziegler (1987) reported that a four-step instructional self-talk plan improved performance in forehand and backhand drive; similarly, Cutton and Landin (2007) found that a five-movement sequence instructional self-talk plan improved forehand drive performance. Despite the fact that the different self-talk cues were used for the accomplishment of one tennis stroke, these studies provided preliminary indirect behavioral evidence for the hypothesis that strategic self-talk helped tennis players shift their attention. The results of the present study further enhance the assumption of the attention-shifting function of strategic self-talk, through the evidence for improved performance in a complex rally, through the shifts within a narrow internal focus of attention.

Considering the conceptual (Hardy et al., 2009) and the evidence-driven (Galanis et al., 2016) models about self-talk mechanisms, based on indirect (e.g., Galanis et al., 2022a; Tzormpatzakis et al., 2022) and direct (e.g., Galanis et al., 2022b) experimental evidence regarding the facilitative effects of strategic self-talk on attention, it can be suggested that strategic self-talk can help shift attentional focus. The current findings are consistent with the relevant literature and reinforce the view that strategic self-talk can facilitate shifting attention in appropriate stimuli equally effective in internal or external modes of attention.

From an applied perspective, the present study demonstrates the application of strategic self-talk in a complex tennis rally based on a ready-made model. This highlights the importance of preparing in training settings that closely resemble real games (Thibodeaux & Winsler, 2020) and the pragmatic view of attentional training (Ziegler, 1987). On the one hand, the findings of this study offer valuable insights for coaches and athletes, particularly regarding the incorporation of psychological techniques into the design and instruction of tennis-specific moves/skills. On the other hand, they underscore the practical relevance of the study, emphasizing the applicability of strategic self-talk in realistic situations and reinforcing its external validity.

Several issues requiring consideration should be noted. To start with, there are two methodological considerations regarding the allocation of the participants and the selection of the self-talk cues. First, participants were recruited from two tennis academies, so the allocation of the participants into groups was not random. Allocating players from the same club into different groups would have probably created greater internal validity problems involving the contamination of the experimental conditions. Thus, it was decided that only the allocation of the groups into experimental and control would be random. Second, the self-talk cues were decided by the experimenters and were assigned to the participant. Hardy (2006) rightly argued that taking a self-determined approach to self-talk cues would assist participants’ sense of autonomy. Nevertheless, it has been noted that when participants do not have the expertise to identify appropriate cues, there is a risk that the cues may not be effective (Hatzigeorgiadis et al., 2020). With that in mind and considering in addition the complexity of the task it was decided that the self-talk cues would be determined by the researchers.

In addition, two more issues related to the interpretation of the results should be noted. First, it is important to note that the attentional interpretation which is suggested was not based on direct evidence, as we did not measure attentional focus, but rather on indirect performance evidence, combined with the attentional demands of the task and the previous literature regarding the attentional functions of self-talk. Second, it should be acknowledged that our postulations regarding the ecological value of the study are of course limited within an experimental framework, as competitive matches involve far more unpredictable and dynamic situations.

Overall, the present study offers valuable evidence regarding the effectiveness of strategic self-talk in a complex tennis performance test that simulates playing conditions. To our knowledge, this is the first study examining the effects of a long self-talk intervention, using a form of narrow internal instructional self-talk plan to investigate the facilitating effect on a test of sequential tennis movements that resemble a competitive situation. The results supported that instructional self-talk can be an effective strategy for shifting attention in body posture with the ultimate goal of improving basic tennis techniques and consequently the entire rally performance.

## 5. Conclusions

In general, the present study offers valuable evidence regarding the effectiveness of strategic self-talk in a complex tennis performance test that simulates playing conditions. To our knowledge, this is the first study examining the effects of narrow internal instructional self-talk among young beginner players. The key aspects of our study were the rather long, considering the relevant literature, self-talk intervention and the use of a multi-stroke performance assessment, which enhances external validity compared with studies using single-act performance tests. The results supported that an internal focus and instructional self-talk can be an effective strategy to improve stroke and rally performance in beginners. Considering the attentional demands of the task, it can be postulated that performance enhancement may have been associated with the efficiency of attention shifting. Further research employing follow-up measures would help identify the sustainability of the performance effects, whereas employing methods to directly assess the shifting of attention, possibly through biomechanical assessment, would further enhance our confidence in the postulated attentional effects.

## Figures and Tables

**Table 1:** Descriptive statistics for the three strokes in the initial and final assessment.

	Initial Assessment				Final Assessment			
	Control		Intervention		Control		Intervention	
	M	SD	M	SD	M	SD	M	SD
Serve	20.40	5.32	17.68	4.84	20.96	3.36	23.26	4.12
Groundstroke	59.12	11.69	60.12	14.26	62.72	11.93	72.80	15.54
Volley	25.40	6.67	25.64	6.10	28.32	7.03	36.84	8.99

## Data Availability

The original contributions presented in this study are included in the article. Further inquiries can be directed to the corresponding author.
